# Supplementary material for: Molecular Alterations in Cutaneous Squamous Cell Carcinoma in Immunocompetent and Immunosuppressed Hosts—A Systematic Review
Source: Cancers (Basel). 2023 Mar 17;15(6):1832. doi: 10.3390/cancers15061832 (PMC10046480; doi:10.3390/cancers15061832)
Supplement: Supplementary file 1 [file cancers-15-01832-s001.zip › cancers-2209624-supplementary.pdf]

SUPPLEMENTARY TABLES

Table S1. MiRNA expression in cSCC. *Abbreviations*: RDEB, Recessive dystrophic epidermolysis bullosa.

MiRNA	Study population	Results	Author	Year
MiR-21	19 specimens consisting of invasive cSCC (*n*=13) and cSCC in situ (*n*=6)	Upregulated in invasive cSCC compared to cSCC in situ (P<0.05)	Stojadinovic et al [32]	2017
MiR-205		Upregulated in invasive cSCC compared to cSCC in situ (P<0.05)		
MiR-31	cSCC cell line (A-431) and normal skin cell line (HaCaT)	miR-31 expression is upregulated in cSCC vs normal skin cell lines (P<0.01). Overexpression of miR-31 significantly increased cell viability and inhibition of miR-31 reduced viability of A-431 cells, P<0.01	Lin et al [33]	2017
MiR-181a	HaCaT, SCC13 and HEK293T cell lines	miR-181a showed low abundance among cSCC compared to normal skin (P=0.0088). miR-181a overexpressing cells grew slower and reach termination criteria at later time points (P=0.0001)	Neu et al [37]	2017
MiR-186	Human cSCC cell line A-431 and the 293-T cell line, 15 paired cSCC and adjacent normal tissues	Invasion and migration were enhanced in A-431 cells transfected with miR-186 mimics and attenuated in A-431 cells transfected with the miR-186 inhibitor compared with the negative control-treated cells (P<0.01)	Tian et al [34]	2018
MiR-221	64 pairs of cSCC tissues and adjacent non-tumor tissues. CSCC cell lines (SCC13, A431, HSC-5 and SCL-1) and HaCaT	miRNA-221 expression was significantly higher in cSCC tissues and cell lines than in normal tissues and cells (P<0.05). Upregulation of miR-221 significantly promoted cell proliferation, while down-regulated expression of miR-221 significantly inhibited cell proliferation	Gong et al [35]	2019
MiR-10b	Cell lines from several donors including RDEB, otherwise healthy, non-RDEB patients, as well as RDEB- and healthy control keratinocytes	Overexpression of miR-10b conferred the stem cell-characteristic of 3D-spheroid formation capacity to keratinocytes. The actin- and tubulin cytoskeleton-associated protein DIAPH2 was identified as a novel putative target of miR-10b	Wimmer et al [31]	2020
MiR-130a	UT-SCC-7 human cSCC cell line, human cSCC tissue samples	MiR-130a expression was almost undetectable in cSCC samples. Overexpression of miR-130a led to a significant reduction in tumor volume by week 4 (P<0.05) and week 5 (P<0.01)	W Lohcharoenkal et al [36]	2021

Table S2. CircRNAs identified with altered expression in cSCC. *Abbreviations*: FC, fold change.

CircRNA	Study population	Results	Author	Year
Circ_EPSTI	9 cSCC and 7 healthy skin samples	Most upregulated in cSCC with a 33-fold increase in expression (P<0.03) compared to healthy skin	Mahapatra et al [39]	2020
Circ_IFFO2 (novel)	9 cSCC and 7 healthy skin samples	Most significantly downregulated circRNA compared to healthy skin (5.3-fold, P<0.0001)		
Circ_KRT1 (novel)	9 cSCC and 7 healthy skin samples	Differentially expressed in cSCC (P=0.00276, FC 0.03RPM)		
Circ_POF1B (novel)	9 cSCC and 7 healthy skin samples	Differentially expressed in cSCC (P=0. 0.009963, fold change 0.06 RPM)		
circ_TNFRSF21 (novel)	9 cSCC and 7 healthy skin samples	4.6-fold upregulated in cSCC compared to healthy skin (P<0.0001)		
hsa_circ_0068631	3 cSCC and 3 non-lesional skin tissues	Upregulated in cSCC vs normal skin (logFC=2.984716944, P=0.008509)	Wei et al [40]	2021
hsa_circ_0070933	3 cSCC and 3 non-lesional skin tissues	Upregulated in cSCC vs normal skin (logFC=2.933784833, P=0.000342)		
hsa_circ_0067772	3 cSCC and 3 non-lesional skin tissues	Upregulated in cSCC vs normal skin (logFC=2.601642222, P=0.00443)		
hsa_circ_0003528	3 cSCC and 3 non-lesional skin tissues	Upregulated in cSCC vs normal skin (logFC=2.59531925, P=0.004158)		
hsa_circ_0070934	3 cSCC and 3 non-lesional skin tissues	Upregulated in cSCC vs normal skin (logFC=2.589680778, P=0.00126)		
hsa_circ_0001955	3 cSCC and 3 non-lesional skin tissues	Upregulated in cSCC vs normal skin (logFC=2.564198778, P=0.001089)		
hsa_circ_0022392	3 cSCC and 3 non-lesional skin tissues	Downregulated in cSCC vs normal skin (logFC=-5.562538167, P=7.57E-08)		
hsa_circ_0022383	3 cSCC and 3 non-lesional skin tissues	Downregulated in cSCC vs normal skin (logFC=-5.410590778, P=3.68E-07)		
hsa_circ_0005085	3 cSCC and 3 non-lesional skin tissues	Downregulated in cSCC vs normal skin (logFC=-4.348301556, P=2.05E-05)		
hsa_circ_0046449	3 cSCC and 3 non-lesional skin tissues	Downregulated in cSCC vs normal skin (logFC=-3.714428278, P=0.00126)		
hsa_circ_0072279	3 cSCC and 3 non-lesional skin tissues	Downregulated in cSCC vs normal skin (logFC=-3.601349444, P=0.000606)		
hsa_circ_0000375	3 cSCC and 3 non-lesional skin tissues	Downregulated in cSCC vs normal skin (logFC=-3.501067111, P=0.000994)		

Table S3. Transcription factors with altered expression in cSCC. *Abbreviations*: C.C, correlation coefficient; KA, keratoacanthoma; PO4-, phosphorylated.

TF	Study population	Results	Author	Year
SMAD 2/3	cSCC (*n*=238), cSCC in situ (*n*=2) and KA (*n*=9) were analysed in comparison with tissues from normal human scalp (*n*=10)	Reduction in active nuclear PO4-SMAD2 and PO4-SMAD3 staining was detected in invasive tumours vs matched perilesional tissue (P<0.001). High-risk tumour depths (≥4mm) demonstrated a highly significant negative dependence on both PO4-SMAD2 (C.C -0.214; P=0.001) and PO4-SMAD3 (C.C -0.200; P=0.002)	Rose et al [41]	2018
E2F1	9 cSCC and 7 healthy skin samples	Overrepresented binding sites among differentially expressed coding genes in cSCC (Z-score 38.02, P-value 0)	Mahapatra et al [39]	2020
ETS1		Overrepresented binding sites among differentially expressed coding genes in cSCC (Z-score 47.28, P-value 0)		
FOXP3		Overrepresented binding sites among differentially expressed coding genes in cSCC (Z-score 47.55, P-value 0)		
Oct-3/4		Overrepresented binding sites among differentially expressed coding genes in cSCC (Z-score 39.09, P-value 0)		
SOX2		Overrepresented binding sites among differentially expressed coding genes in cSCC (Z-score 36.77, P-value 0)		

Table S4. LncRNA expression in cSCC. *Abbreviations*: FC, fold change; FDR, false discovery rate; Lce, late cornified envelope.

LncRNA	Study population	Results	Author	Year
AK144841	Mouse skin	AK144841 expression was 40-fold higher in cSCC than in healthy skin. AK144841 downregulated the expression of genes of the Lce1 family	Ponzio et al [44]	2017
LINC00319	60 paired cSCC and matched normal skin samples. HaCaT, 4 cSCC cell lines (A431, HSC-5, SCC13, and SCL-1)	LINC00319 overexpression accelerated SCL-1 cell proliferation compared with empty control group, whereas LINC00319 knockdown slowed A431 cell proliferation (P<0.01)	Li et al [46]	2018
TINCR	cSCC cell line A431	TINCR overexpression promoted apoptosis induced by ALA-PDT and silencing TINCR inhibited apoptosis (P<0.05)	Zhou et al [51]	2018
LINC00520	Human cSCC cell line A431	Downregulated in cSCC	Mei et al [50]	2019
LINC01048	80 pairs of human CSCC samples and adjacent non-tumorous tissues. Human cSCC cell lines (SCC13 and SCL-1), HaCaT	LINC01048 was upregulated in cSCC compared with adjacent noncancerous tissues (P<0.01). Mechanistically, LINC01048 was transcriptionally activated by *USF1*	Chen et al [45]	2019
HOTAIR	HaCaT and 4 cSCC cell lines: A431, HSC-5, SCC13, and SCL-1	Level of HOTAIR in cSCC was increased remarkably vs HaCaT (P<0.05). Downregulation of HOTAIR decreased the tumor volume and tumor weight (P<0.05)	Yu et al [49]	2019
MALAT1	cSCC lines (A431, HSC-1, and HSC-5), HaCaT	MALAT1 knockdown drastically inhibited A431 and HSC-1 cell invasiveness (P<0.001)	Y. Zhang et al [43]	2019
KB-1410C5.3/lnc-GRHL2	9 cSCC and 7 healthy skin samples	Most downregulated lncRNA (0.005-fold compared to healthy skin, FDR 8.95E-29)	Mahapatra et al [39]	2020
RP11-493L12.5		Most upregulated lncRNA in cSCC (46.77-fold compared to healthy skin, FDR 4.05E-16)		
EZR-AS1	66 cSCC tissues and healthy adjacent non-cancerous tissues. CSCC cell lines (SCL-1, SCC13, A431 and HSC-5) and HaCaT	EZR-AS1 mRNA expression levels were significantly upregulated in cSCC compared with adjacent healthy tissues and HaCaT cells (P<0.01) EZR-AS1 knockdown inhibited cSCC cell migration and invasion, and promoted cell apoptosis	Lu et al [47]	2020
HCP5	cSCC and healthy skin tissue controls from 60 patients CSCC cell lines (A431, COLO-16, SCC13, SCL-1, HSC-1, and HSC-5) and HaCaT	HCP5 had the greatest upregulation in cSCC (logFC=1.8) and the highest relative expression levels in cSCC vs normal adjacent tissue (P<0.001). Silencing of HCP5 expression resulted in significant decreases in A431 cell viability, invasion (P<0.001) and migration (P<0.01) vs normal tissue	Zou et al [48]	2021
AL353997.3	Paired human cSCC and normal skin samples	Differentially expressed in cSCC (threshold of FC ≥2.0 and P≤0.05). Upregulated in cSCC vs normal skin	Hu et al [42]	2022
BX004987.5		Differentially expressed in cSCC (threshold of FC ≥2.0 and P≤0.05). Downregulated in cSCC vs normal skin		
CTD-2521M24.9		Differentially expressed in cSCC (threshold of FC ≥2.0 and P≤0.05). Upregulated in cSCC vs normal skin		
CTD-2619J13.13		Differentially expressed in cSCC (threshold of FC ≥2.0 and P≤0.05). Downregulated in cSCC vs normal skin		
LINC00478		Differentially expressed in cSCC (threshold of FC ≥2.0 and P≤0.05). Downregulated in cSCC vs normal skin		
MIR4720		Differentially expressed in cSCC (threshold of FC ≥2.0 and P≤0.05). Downregulated in cSCC vs normal skin		
PVT1		Differentially expressed in cSCC (threshold of FC ≥2.0 and P≤0.05). Upregulated in cSCC vs normal skin		
LINC01003	Matched tumor and blood DNA from 25 patients with regional metastases of cSCCHN	Significant functional alterations were observed in the tumor suppressing lncRNA LINC01003 (68% of specimens, Q-value: 0.0158)	Thind et al [16]	2022

Table S5. Proteins downregulated in cSCC. *Abbreviations*: TP/PP, cancer tissues (TPs) and noncancerous tissues (PPs) ratio.

Protein	Study population	Results	Author	Year
β-catenin	85 human epidermal resection specimens: normal (*n*=42), pre-cancerous (*n*=34) and cSCC (*n*=51)	Membrane β-catenin expression significantly reduced from normal to SCC (93% to 69%, P<0.001)	Sun et al [52]	2019
CK10		Inversely correlated with cancer development (rs=-0.626, P<0.001)		
COL1A1	20 pairs of primary human cSCC and matched noncancerous tissue samples	Decreased in cSCC, mapped to protein digestion and absorption and platelet activation pathways (TP/PP=0.24). Also connected to focal adhesion pathway	W. Chen et al [53]	2021
COL28A1		Decreased in cSCC, mapped to protein digestion and absorption pathway (TP/PP=0.06)		
COL6A6		Decreased in cSCC, mapped to protein digestion and absorption pathway (TP/PP=0.08). Also connected to focal adhesion pathway		
TLN2		Decreased in cSCC, mapped to platelet activation pathway (TP/PP=0.51). Also connected to focal adhesion pathway		

Table S6. Proteins upregulated in cSCC. *Abbreviations*: AK, actinic keratosis; BD, Bowen disease; FC, fold change; WT, wild-type.

Protein	Study population	Results	Author	Year
ALB	Human normal epidermis (n=4, pooled), AK (*n*=10), BD (*n*=10) and cSCC (*n*=10)	Differentially abundant in cSCC compared to AK (Fold change 19.9, adjusted P<0.05; n ≥8)	A. Azimi et al [54]	2018
APOA1		Differentially abundant in cSCC compared to AK (FC=5.3, adjusted P<0.05; n ≥8)		
FLNA		Differentially abundant in cSCC compared to normal skin – unique to cSCC vs AK and BD (FC=6.8, adjusted P<0.05; n ≥8)		
FSCN1		Differentially abundant in cSCC compared to normal skin – unique to cSCC vs AK and BD (FC=4.2, adjusted P<0.05; n ≥8)		
HLA-B		Differentially abundant in cSCC compared to AK (FC=5.2, adjusted P<0.05; n ≥8)		
HP		Differentially abundant in cSCC compared to AK (FC=4.8, adjusted P<0.05; n ≥8)		
IGHA1		Differentially abundant in cSCC compared to normal skin – unique to cSCC vs AK and BD (FC=5.4, adjusted P<0.05; n ≥8)		
LGALS1		Differentially abundant in cSCC compared to normal skin – unique to cSCC vs AK and BD (FC=4.3, adjusted P<0.05; n ≥8)		
MAP4		Differentially abundant in cSCC compared to normal skin – unique to cSCC vs AK and BD (FC=4.9, adjusted P<0.05; n ≥8)		
SERPINA1		Differentially abundant in cSCC compared to AK (FC=6.9, adjusted P<0.05; n ≥8)		
TXNDC5		Differentially abundant in cSCC compared to AK (FC=4.4, adjusted P<0.05; n ≥8)		
IGF2BP	Human cSCC tissues from 9 patients, surrounding normal skin samples. A431 human cSCC cell line	IGF2BP1 knockout significantly inhibited A431 cell survival and proliferation (P<0.05)	Z. Liu et al [58]	2018
CD44	85 human epidermal resection specimens: normal (*n*=42), pre-cancerous (*n*=34) and cSCC (*n*=51)	Positively stained in 17% of normal tissues vs 61% in SCC, P<0.001. Positive correlation with cancer development, rs=0.383 (P<0.001)	Sun et al [52]	2019
CK17		Positively correlated with cancer development (rs=0.67, P<0.001). Positively stained in 9% of normal tissues vs 82% in SCC		
E-cadherin		Positively stained in 98% of normal tissues vs 92% in SCC, P<0.001		
EXOSC10		Expression increased from normal to cSCC (14% to 65%, P<0.001). EXOSC10 staining was positively correlated with disease development (rs=0.392, P<0.001)		
EZR		Positively stained in 21% of normal tissues vs 96% in SCC, P<0.001. Positive correlation with cancer development, rs=0.717 (P<0.001)		
Hsp75		Expression increased from 7% in normal skin to 73% in cSCC (P<0.001)		
Hsp90-α		Expression increased from 29% in normal skin to 100% in SCC (P<0.001)		
SOD2		Positively stained in 2% of normal tissues vs 63% in SCC (P<0.001)		
CMG2	HPV38E6E7 SCC cell line	CMG2 was significantly overexpressed in tumor tissue, with detectable expression in the UV-control	Crawford et al [56]	2019
TEM8		TEM8 was significantly overexpressed in tumor tissue compared to UV-control		
Cox-2	Hair follicle stem cell-originating cSCC wild-type Cox-2 (*n*=15), Cox-2 knockout (*n*=11)	WT Cox-2 tumors frequently demonstrated mesenchymal-like spindle cell carcinomas with minimal keratinization (11 of 15). Cox-2 KO tumors were often well-differentiated with significant hyperkeratosis or papillomatous growths (6 of 11).	H Moon et al [55]	2020
LPCAT1	43 human cSCC samples	LPCAT1 is upregulated in cSCC samples and cell lines vs primary human epidermal keratinocytes (P<0.001). LPCAT1 depletion increased both the early and late apoptosis rates, with the total rate approaching 12-19% vs 3-4% in controls	Y Huang et al [57]	2021
PIK3CB	20 pairs of primary human cSCC and matched noncancerous tissue samples	Elevated in cSCC, mapped to platelet activation pathway (TP/PP=4.11)	W. Chen et al [53]	2021

Table S7. Proteins with roles in cSCC metastasis and differentiation. *Abbreviations*: FC, fold change; MET, patients with lymph node metastases, but with no available primary tumor; NHEK, normal human epidermal keratinocyte; NS, normal healthy skin; RDEB, Recessive dystrophic epidermolysis bullosa; SES, sun-exposed skin; Padj, adjusted P-value; PRI-, locally confined tumors; PRI+, primary tumors that had metastasized; TAp63−/−, homozygous deletion of TAp63; WT, wild-type.

Protein	Study population	Results	Author	Year
ΔNp63	Genetically engineered mouse models: Lgr5CreER and K14CreER mice	p63 was only expressed in tumor epithelial cells (TECs) and not in tumor mesenchymal-like cells (TMCs). Proportion of well-differentiated SCCs was strongly increased in tumors that expressed ΔNp63 (P=0.0009)	Latil et al [26]	2017
LGALS3BP	Human cSCC: low-risk (*n*=10), metastasizing (*n*=10), RDEB cSCC (*n*=10)	Significantly increased abundance of LGALS3BP in high-risk cSCC vs low-risk cSCC (P<0.05)	Föll et al [60]	2018
MARCKS		MARCKS was present in both groups of high-risk cSCC and virtually absent in low-risk cSCC (P<0.01)		
PABPC1		Staining for PABPC1 revealed strong presence in cancer keratinocytes of both high-risk cSCC compared to low-risk cSCC (P<0.05)		
RAC1		In metastasizing cSCC, RAC1 showed clear membrane-associated staining of individual cSCC keratinocytes within tumor islands. Staining was significantly less intense in low-risk cSCC (P<0.05)		
SND1		SND1 was significantly increased in both groups of high-risk cSCC and compared to low-risk cSCC (P<0.05)		
iASPP	116 human cSCC samples, 10 human cSCC cell lines	Poorly differentiated cSCC displayed significantly higher cytoplasmic and lower nuclear iASPP expression (high nuclear iASPP expression found in 48% of well-differentiated cSCC vs 4.2% in poorly differentiated cSCC)	DJ Robinson et al [62]	2019
TAp63	5 human cSCC cell lines (COLO16, SRB12, SRB1, IC1, and RDEB2), NHEK, mouse models	Higher frequency of cSCC in TAp63−/− cohort vs WT (46.67% vs 20%). No metastases were found in WT mice, whereas multiple lung metastases were observed in TAp63−/− mice with cSCC	Davis et al [63]	2020
APCS	Primary (*n*=20) and metastatic cSCC (*n*=25) samples	Decreased in metastatic cSCC compared to the primary lesions (Padj=6.S0E-04, FC=-7.1)	A. Azimi et al [59]	2020
APOA1		Increased in metastatic cSCC compared to the primary lesions (Padj=4.79E-02, FC=2.5)		
CST6		Decreased in metastatic cSCC compared to the primary lesions (Padj=2.57E-02, FC=-3.9)		
DMKN		Decreased in metastatic cSCC compared to the primary lesions (Padj=6.S0E-04, FC=-9.4)		
ISG15		Increased in metastatic cSCC compared to the primary lesions (Padj=3.33E-02, FC=2.7)		
MARCKS		Increased in metastatic cSCC compared to the primary lesions (Padj=3.91E-02, FC=1.9)		
ENTPD1 (CD39)	Human cSCC, unmatched NS	Human cSCC tumors displayed elevated levels of ENTPD1 mRNA and ENTPD1 protein expression vs unmatched NS (*n*=8 for NS, *n*=10 for cSCC, P<0.05). ENTPD1 expression is significantly higher in human cSCC that metastasize than in those that are nonmetastatic ([+] Met, *n*=54, [-] Met, *n*=51, P<0.001)	MJ Whitley et al [61]	2021
uPAR	cSCCHN from 50 patients. 21 PRI-, 14 PRI+, 15 MET, matched SES	Significantly increased uPAR staining in MET tissues compared to PRI- (P<0.0001)	Minaei et al [7]	2022

Table S8. Metabolites mapped to regulatory pathways in cSCC.

Metabolite	Study population	Results	Author	Year
L-Glutarnate	20 pairs of primary human cSCC and matched noncancerous tissue samples	Elevated in cSCC, mapped to protein digestion and absorption pathway (TP/PP=3.70)	W. Chen et al [53]	2021
L-Arginine	20 pairs of primary human cSCC and matched noncancerous tissue samples	Elevated in cSCC, mapped to protein digestion and absorption pathway (TP/PP=2.43)		
L-Aspartate	20 pairs of primary human cSCC and matched noncancerous tissue samples	Elevated in cSCC, mapped to protein digestion and absorption pathway (TP/PP=3.40)		
L-Glutamine	20 pairs of primary human cSCC and matched noncancerous tissue samples	Elevated in cSCC, mapped to protein digestion and absorption pathway (TP/PP=2.40)		
L-Phenylalanine	20 pairs of primary human cSCC and matched noncancerous tissue samples	Elevated in cSCC, mapped to protein digestion and absorption pathway (TP/PP=2.01)		
Arachidonate	20 pairs of primary human cSCC and matched noncancerous tissue samples	Decreased in cSCC, mapped to platelet activation pathway (TP/PP=0.24)		
